# Le syndrome d´encéphalopathie postérieure réversible en cas de pré-éclampsie: à propos d´un cas

**DOI:** 10.11604/pamj.2022.43.1.33548

**Published:** 2022-09-01

**Authors:** Rania Damak, Salma Ketata, Rahma Derbel, Faiza Grati, Sahar Ghorbel, Imen Zouche, Zied Triki

**Affiliations:** 1 Service d´Anesthésie Réanimation, Centre Hospitalier Universitaire Habib Bourguiba Sfax, Sfax, Tunisie

**Keywords:** Le syndrome d’encéphalopathie postérieure réversible, éclampsie, femme enceinte, cas clinique, Reversible posterior encephalopathy syndrome, eclampsia, pregnant woman, case report

## Abstract

Le syndrome d´encéphalopathie postérieure réversible est un syndrome clinico-radiologique rare. Ce diagnostic est suspecté, chez la femme enceinte souffrante d´éclampsie, lorsque le scanner cérébral révèle des images radiologiques évocatrices de cette pathologie. Nous rapportons le cas d´une patiente âgée de 25 ans. Elle était enceinte au terme de 33 semaines d´aménorrhée. Elle était pré-éclamptique. Suite à son accouchement par césarienne, elle a présenté des crises convulsives répétitives et des chiffres tensionnels élevés. La patiente a séjourné en réanimation; les explorations clinico-radiologiques ont objectivé: un syndrome d´encéphalopathie postérieure réversible. La patiente a bénéficié durant son séjour d´un contrôle de la pression artérielle et d´un traitement anti convulsivant. L´évolution clinique a été favorable. Le syndrome d´encéphalopathie postérieure réversible est une manifestation neurologique survenant rarement au cours de la pré-éclampsie, mais non exceptionnelle, donc il faut y penser devant tout signe neurologique. L´imagerie par résonnance magnétique (IRM) cérébrale est le meilleur outil diagnostique.

## Abstract (en)

Reversible posterior encephalopathy syndrome (PRES) is a rare clinico-radiological syndrome. Diagnosis is suspected in pregnant women suffering from eclampsia when brain CT scan shows radiological images suggestive of this disease. We here report the case of a 25-year-old pregnant woman at 33 weeks’ gestation with a history of pre-eclampsia presenting with convulsive seizures and high blood pressure following cesarean section. The patient was admitted to the Intensive Care Unit; clinical and radiological investigations revealed PRES syndrome. The clinical course was favorable after control of blood pressure by appropriate treatment and anticonvulsant therapy. Reversible posterior encephalopathy syndrome is a neurological manifestation rarely occurring in patients with pre-eclampsia, but it is not exceptional; then diagnosis should be suspected in patients with neurological signs. Brain MRI is the best diagnostic tool.

## Introduction

Le syndrome d´encéphalopathie postérieure réversible est un syndrome clinico-radiologique rare. Ce diagnostic est évoqué chez des patients présentant des symptômes neurologiques comme: des céphalées, une confusion, des troubles visuels, et/ou une crise d´épilepsie. Ces symptômes se manifestent dans un contexte d´insuffisance rénale, d´hypertension artérielle, de certaines maladies auto-immune, pré-éclampsie ou d´éclampsie. A ces signes cliniques s´associent des signes scannographiques. Il est confirmé par l´imagerie par résonnance magnétique (IRM).

## Patient et observation

**Présentation du patient**: nous rapportons le cas d´une patiente âgée de 25 ans, primipare, primigeste et enceinte de 33 semaines d´aménorrhée. Elle n´avait pas d´antécédents médicaux particuliers. Sa grossesse était compliquée de pré-éclampsie. Ce diagnostic a été retenu suite à des chiffres tensionnels élevés.

**Résultats cliniques et chronologie**: la patiente a présenté une crise convulsive tonico clonique à son domicile. A son arrivée à l´hôpital, la patiente était consciente. L´examen neurologique était normal. Sa tension artérielle était à 170/99 mm de Hg. Son bilan biologique était normal. Une extraction fœtale en urgence par césarienne, sous nécessitant son intubation et sa mise sous ventilation mécanique rachianesthésie a été faite. A H 8 post opératoire, la patiente a présenté une deuxième crise d´éclampsie. Elle a été transférée au service de réanimation chirurgicale. A son admission, la patiente était intubée, ventilée, et sédatée. Ses pupilles étaient en myosis. Ses réflexes ostéotendineux étaient présents mais non vifs. Ses réflexes cutanéo-plantaires étaient indifférents des deux côtés. Son pouls était à 100 battements par minutes. Sa diurèse horaire était conservée à 150 cc/h. Ses mollets étaient souples. Elle avait un bon globe utérin. Le bilan biologique à l´admission était correcte: elle n´avait pas d´anémie, ni de thrombopénie. Elle n´avait ni de cytolyse hépatique, ni de troubles ioniques. Sa tension artérielle était inférieure à 140/90 mm Hg sous nicardipine pure à la pousse-seringue-électrique. Un traitement par sulfate de magnésium a été débuté.

**Figure 1:**
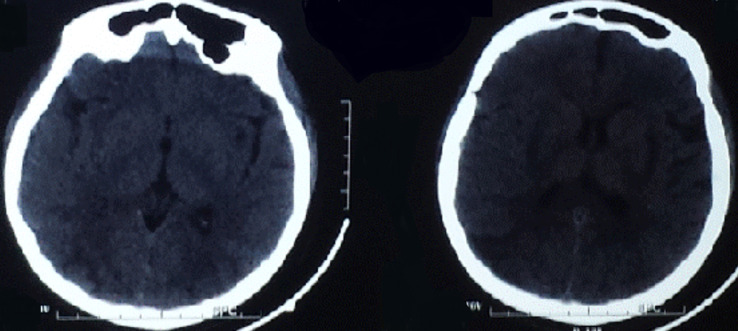
coupe scannographique cérébrale (quelques plages hypo denses mal limitées cortico sous corticales postérieures pariéto-occipitale, temporale droite et capsulaire externe gauche, non rehaussée après injection de produit de contraste)

**Figure 2:**
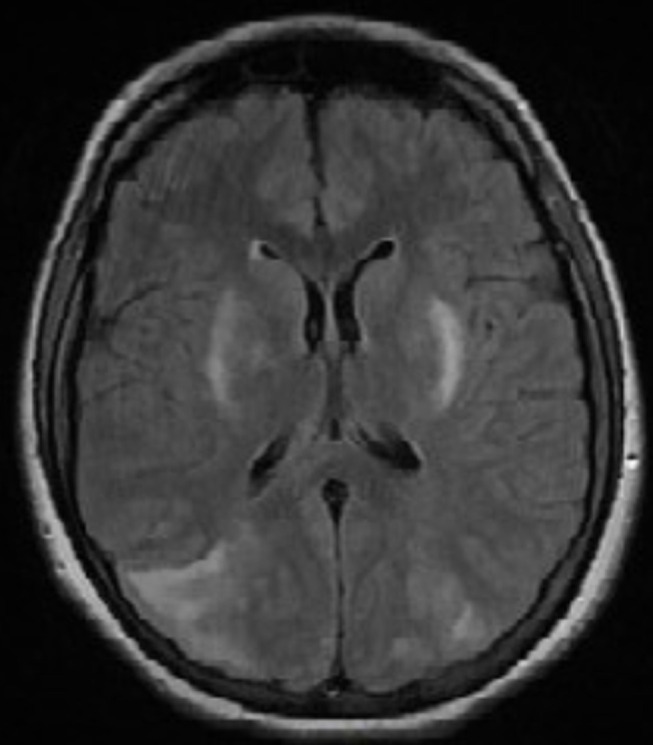
coupe IRM cérébrale T2 flair (hypersignal T2 cortico sous cortical pariéto occipital prédominant à droite)

**Démarche diagnostique**: un scanner en urgence a été fait. Il a montré quelques plages hypo denses, mal limitées, cortico sous corticales, postérieures pariéto-occipitale, temporale droite et capsulaire externe gauche, non rehaussée après injection de produit de contraste: c´est un aspect en faveur d´un syndrome d´encéphalopathie postérieure réversible (Figure 1). Le diagnostic a été confirmé par une IRM cérébrale qui a montré: un hyper signal T2 cortico-sous-cortical, globalement symétrique prédominant à droite, sans hémorragie au niveau pariéto-occipital postérieur et frontal supérieur bilatéral. Il existe une anomalie du signal capsulo- lenticulaires symétrique bilatérale sans restriction de la diffusion. La séquence de diffusion trouve un petit œdème cytotoxique, occipital, bilatéral de taille centimétrique (Figure 2).

**Intervention thérapeutique**: la patiente a été intubée au service de gynéco obstétrique. En unité de réanimation, un traitement par sulfate de magnésium et par nicardipine à la pousse-seringue-électrique a été instauré. Notre objectif de tension artérielle était d´atteindre des valeurs inférieures à 140 /90 mm de Hg. De la dexaméthasone et du clonazépam ont été aussi prescrits à cette patiente.

**Suivi et résultats des interventions thérapeutiques**: l´évolution clinique était favorable. La patiente était extubée à j2 post opératoire. Son examen neurologique était normal. Elle était transférée au service de gynéco-obstétrique après 3 jours d´hospitalisation en unité de réanimation.

**Consentement éclairé**: il a été obtenu de la patiente après l´avoir informée.

## Discussion

Le syndrome d´encéphalopathie postérieure réversible ou PRES Syndrome est une unité clinico-radiologique qui était définie depuis une vingtaine d´années par l´équipe de Hinchey *et al*.[1]. Ce diagnostic était évoqué principalement chez des patients présentant des symptômes neurologiques aiguës ou subaigües qui étaient le plus souvent: des céphalées, des troubles visuels et ou une crise convulsive isolée. Ces symptômes étaient associés le plus souvent à une insuffisance rénale, une hypertension artérielle, certaines maladies auto-immunes, une pré-éclampsie ou une éclampsie [2]. Les symptômes neurologiques les plus fréquents sont des céphalées. Elles sont généralement diffuses est d´installation progressive. En absence de prise en charge, les symptômes s´aggravent progressivement sur plusieurs jours à plusieurs semaines. Ils peuvent aller jusqu´à: l´encéphalopathie, la confusion, les convulsions, et même le coma [3]. Il existe également des formes graves avec hémorragie ou œdème massif de la fosse postérieure entraînant une hydrocéphalie ou compression du tronc cérébral [2].

Radiologiquement, l´IRM cérébrale est le *gold standard* pour le diagnostic du syndrome d´encéphalopathie postérieure réversible. Bien que le scanner garde une place pour éliminer un accident hémorragique, il peut évoquer ce syndrome dans 50% des cas [4]. Ce désordre apparait au scanner comme une hypodensité au niveau de la fosse postérieure et des lobes occipitaux. L´IRM montre une hyperdensité T2 et Flair et en hyposignal T1. Parfois la séquence T2 ne peut distinguer l´œdème vasogénique, qui est réversible, de l´œdème cytotoxique, qui est irréversible. Ils peuvent être différentiés seulement par diffusion [5,6]. Parfois une petite hémorragie intracrânienne survient près des lésions radiologiques de ce syndrome [7]. L´anomalie la plus communément observée est l´œdème cérébral sans infarctus. Elle touche typiquement de façon bilatérale et symétrique la substance blanche sous corticale dans les régions postérieures des hémisphères cérébraux, et en particulier les régions pariéto-occipitales[8,9]. Il existe, parfois, une distribution atypique des lésions: atteintes non cortico-sous-corticales de la substance blanche profonde (capsule interne, externe, centre semi ovale, corps calleux) et des noyaux gris centraux. L´atteinte de la substance blanche est constante mais la substance grise n´est affectée que dans 30% des cas [10].

Les lésions perçues radiologiquement traduisent l´œdème cytotoxique et vasogénique. La physiopathologie de cet œdème est discutable. Dans la littérature, on trouve deux théories: la première théorie est celle de l´hyper perfusion cérébrale: en effet, l´hypertension artérielle dépasse la capacité d´autorégulation du cerveau et entraine une altération vasculaire et une vasodilatation artériolaire. La rupture de la barrière hématoencéphalique qui apparaît secondairement est à l´origine d´une fuite liquidienne des vaisseaux vers le parenchyme cérébral et donc d´un œdème vasogénique réversible [11]. La deuxième théorie est celle de l´hypoperfusion cérébrale qui est secondaire à l´hypertension artérielle ou à un processus systémique. Elle peut être observée dans la pré-éclampsie, les infections, la chimiothérapie. En effet, lorsque le système immunitaire est activé, les cellules endothéliales seront ensuite lésées, d´où l´œdème cytotoxique [12]. En cas de syndrome d´encéphalopathie postérieure réversible, le but de la thérapeutique est de: contrôler la pression artérielle et les convulsions, et de réduire le risque de vasospasme, d´hémorragie et de thrombose veineuse [13].

Le contrôle de l´HTA est le volet primordial du traitement. Il fait appel aux agents antihypertenseurs habituels comme les inhibiteurs calciques (nicardipine ou diltiazem), les bêtabloquants (labétolol notamment) et les diurétiques. L´objectif thérapeutique est de maintenir une pression artérielle moyenne entre 105 et 125mmHg, sans réduire cette pression de plus de 25% durant la première heure [1,8]. Le sulfate de magnésium (MgSO4) est à la fois un antihypertenseur, et a un effet protecteur contre les convulsions. Il est primordial d´instaurer ce traitement en cas de pré-éclampsie ou d´éclampsie. Le MgSO4 a aussi une action protectrice de la barrière cérébrale, donc prévient l´œdème cérébral. Il réduit aussi la vasoconstriction cérébrale et périphérique et diminue les résistances vasculaires [14]. Les agents anti épileptiques ont aussi une place dans le traitement de ce syndrome. En cas de crise convulsive, un traitement antiépileptique doit être instauré en urgence. Les benzodiazépines (clonazépam ou diazépam) doivent être administrées en première ligne par voie intraveineuse.

En seconde ligne ou en cas d´état de mal, il faut recourir à la phénytoïne ou au phénobarbital. L´acide valproïque est une option thérapeutique, notamment en cas d´insuffisance cardiaque, chez les personnes âgées. En cas d´état de mal réfractaire, les agents de choix sont le propofol, le midazolam et le thiopental. Le traitement anti-œdémateux (mannitol) et l´administration de corticoïdes doivent être discutés au cas par cas et peuvent être bénéfiques dans certaines situations [8]. Pour notre cas; la patiente était sédatée par midazolam et fentanyl pendant deux jours, elle a eu une dose de charge et une dose d´entretien de MgSO4. Elle était aussi mise sous nifédipine. Une corticothérapie par dexaméthasone a été aussi instaurée. Au réveil, la patiente n´a pas montré de déficit neurologique. Les crises convulsives n´ont pas récidivé. Habituellement, l´évolution en cas de syndrome d´encéphalopathie postérieure réversible est favorable sous traitement adapté. Il y a une résolution des signes cliniques au bout de 3 à 8 jours. Mais dans 5 à 12 % des cas, l´évolution peut être défavorable avec persistance de séquelles neurologiques ou même peut conduire au décès [15,16].

## Conclusion

Le syndrome d´encéphalopathie postérieure réversible est une manifestation neurologique survenant rarement au cours de la pré-éclampsie. Il faut y penser devant tout signe neurologique chez la femme enceinte. L´IRM en séquence de diffusion est le meilleur outil diagnostique, notamment pour écarter un accident ischémique cérébral.
